# Neuroprotective Effect of Ginkgolide B on Bupivacaine-Induced Apoptosis in SH-SY5Y Cells

**DOI:** 10.1155/2013/159864

**Published:** 2013-10-21

**Authors:** Le Li, Qing-guo Zhang, Lu-ying Lai, Xian-jie Wen, Ting Zheng, Chi-wai Cheung, Shu-qin Zhou, Shi-yuan Xu

**Affiliations:** ^1^Department of Anesthesiology, Zhujiang Hospital, Southern Medical University, Guangzhou, Guangdong 510282, China; ^2^Department of Anesthesiology, The University of Hong Kong, Hong Kong

## Abstract

Local anesthetics are used routinely and effectively. However, many are also known to activate neurotoxic pathways. We tested the neuroprotective efficacy of ginkgolide B (GB), an active component of Ginkgo biloba, against ROS-mediated neurotoxicity caused by the local anesthetic bupivacaine. SH-SY5Y cells were treated with different concentrations of bupivacaine alone or following preincubation with GB. Pretreatment with GB increased SH-SY5Y cell viability and attenuated intracellular ROS accumulation, apoptosis, mitochondrial dysfunction, and ER stress. GB suppressed bupivacaine-induced mitochondrial depolarization and mitochondria complex I and III inhibition and increased cleaved caspase-3 and Htra2 expression, which was strongly indicative of activation of mitochondria-dependent apoptosis with concomitantly enhanced expressions of Grp78, caspase-12 mRNA, protein, and ER stress. GB also improved ultrastructural changes indicative of mitochondrial and ER damage induced by bupivacaine. These results implicate bupivacaine-induced ROS-dependent mitochondria, ER dysfunction, and apoptosis, which can be attenuated by GB through its antioxidant property.

## 1. Introduction

Local anesthetics are among the most common clinical drugs and are generally regarded as safe [1, 2]. However, they have also been shown to be neurotoxic even at normal clinical dose [3, 4]. This neurotoxicity is mediated at least in part by activation of apoptotic pathways [5, 6]. In the cauda equina, intrathecally administered local anesthetics induced cell swelling, atrophy, edema, axonal degeneration, and the appearance of myelin ovoids as well as macrophage infiltration [7]. These morphological signs of degeneration indicate that local anesthetics can initiate a complex cascade of direct cytotoxic and ensuing inflammatory responses, although the molecular mechanisms of local anesthetic toxicity are still largely unknown.

Local anesthetics have been shown to induce neural dysfunction and apoptosis *in vitro *[8–11]. For example, bupivacaine may inhibit mitochondrial respiratory complexes I and III, leading to decreased ATP production, collapse of the mitochondrial membrane potential (Δ*ψ*m), overproduction of reactive oxygen species (ROS), and ultimately liberation of cytochrome c and activation of the caspase-3-dependent apoptosis pathway [10–12]. In fact, ROS accumulation, mitochondrial uncoupling, and depolarization of *ψ*m are among the earliest indicators of apoptosis induced by local anesthetics [13, 14]. In addition to mitochondrial damage, dysfunction of the endoplasmic reticulum (ER) stress has also been implicated in apoptosis. Arai and Nonaka et al. proposed that oxidative stress associated with local anesthetics can induce Ca^2+^ release from intracellular stores, including the rough endoplasmic reticulum (rER) [11, 15]. Loss of intraluminal Ca^2+^ may lead to ER stress [16], further, ROS generation, [17] and activation of ER-dependent apoptosis pathways [18]. Thus, mitochondrial and ER damage associated with ROS overproduction may act synergistically to evoke cell death in response to bupivacaine or other structurally related local anesthetics.

Ginkgo biloba has been used in traditional Chinese medicine for thousands of years. Evidence accumulated over the last decade suggests that concentrated and partially purified extracts of Ginkgo biloba leaves may afford protection against certain neurological diseases [19]. Indeed, ginkgolide B (GB), the major active component of Ginkgo biloba extract, has been used to treat degenerative dementia and neurosensory disorders [20]. Even for children, GB is a safe drug without adverse reactions [21]. Furthermore, GB reduced the level of ROS *in vivo*, suggesting that the raw extract contains antioxidants [22]. Therefore, GB may protect neurons against the neurotoxicity of local anesthetics like bupivacaine by reducing oxidative stress.

The principal aims of this study were to examine the molecular mechanisms of bupivacaine toxicity and the neuroprotective efficacy of GB *in vitro*.

## 2. Materials and Methods

### 2.1. Materials

The human neuroblastoma cell line SH-SY5Y was purchased from the Shanghai Institutes for Biological Sciences. Bupivacaine hydrochloride (purity 99.9%) was purchased from Sigma (St. Louis, MO, USA). Ginkgolide B (purity ≥ 99.5%) was obtained from the National Institutes for Food and Drug Control and dissolved in dimethyl sulfoxide (DMSO) (KeyGEN, China). Other reagents used included DMEM/F12 medium and fetal bovine serum (Gibco, USA), 5,5′, 6,6′-tetrachloro-1,1′, 3,3′-tetraethyl tetrethyl benzimidalyl carbocyanine iodide (JC-1), 3-(4,5-dimethyl-2-thiazolyl)-2,5-diphenyl-2-tetrazolium bromide (MTT), 2′,7′-dichlorofluorescein diacetate (DCFH-DA), mitochondrial isolation agent and mitochondrial storage solution (all from Beyotime, China), anti-Grp78 and anti-caspase-12 (Abgent, USA), anti-cleaved caspase-3 and anti-HtrA2 (Abcam, UK), anti-GAPDH antibody (Goodhere, China), and Annexin V-FITC and propidium iodide (KeyGEN, China). The cell counting Kit-8 (CCK8) was purchased from Dojindo (Dojindo, Kumamoto, Japan). All reagents were obtained from commercial suppliers and were of standard biochemical quality.

### 2.2. Cell Culture

Cells of the SH-SY5Y line were cultured in DMEM/F12 medium supplemented with 15% fetal bovine serum, 100 U/mL penicillin, and 100 *μ*g/mL streptomycin and maintained in a humidified 5% CO_2_ incubator at 37°C. The media were changed every 2 days.

### 2.3. MTT Assay

The effect of bupivacaine on the number of viable SH-SY5Y cells was determined by the MTT assay. Cells were seeded onto 96-well plates at 5 × 10^3^ cells/well with 100 *μ*L of culture medium and treated with various concentrations of bupivacaine as indicated below. Treated cells were incubated with 20 *μ*L MTT at 37°C for 4 h, the medium removed, and 150 *μ*L DMSO added to dissolve the formazan crystals produced from MTT by viable cells. The optical density (OD) of the homogenous purple formazan/DMSO solutions was measured using a spectrophotometer (Bio-Tek, USA) at 570 nm.

### 2.4. CCK-8 Assay

Cells were seeded onto 96-well plates at 5 × 10^3^ cells/well in 100 *μ*L culture medium. In pilot experiments to determine the working range of GB, cultures were pretreated with 5–40 *μ*mol/L GB in new media for 6 h or subjected to a control media change prior to treatment with 1 mmol/L bupivacaine for 24 h (the half-maximal neurotoxic dose according to [23]). After bupivacaine treatment, 10 *μ*L of CCK-8 was added to each well for another 3 h at 37°C. The OD was read at 450 nm using a spectrophotometer.

### 2.5. Apoptosis Assay by Flow Cytometry

Cells were seeded onto 24-well plates at 5 × 10^5^ cells/well in 500 *μ*L culture medium. After control or GB pretreatment and bupivacaine administration as described, the cells were rinsed with PBS, harvested, and resuspended in 500 *μ*L binding buffer. To this cell suspension was added 5 *μ*L Annexin V-FITC (a marker of early apoptosis) and 5 *μ*L propidium iodide (a marker of late apoptosis). After 10 min incubation, cell apoptosis was determined by flow cytometry (BD FACS Calibur, USA).

### 2.6. Measurement of Reactive Oxygen Species

Cells were seeded onto 24-well plates at 5 × 10^5^ cells/well in 500 *μ*L culture medium and divided into four treatment groups: (i) untreated controls (Con), (ii) cells treated with 1 mmol/L bupivacaine for 24 h (Bup), (iii) cells pretreated with 40 *μ*mol/L GB for 6 h, and (iv) cells treated with 40 *μ*mol/L GB for 6 h prior to 1 mmol/L bupivacaine exposure for 24 h (GB + Bup). Intracellular accumulation of ROS was estimated using the redox-sensitive fluorescent dye DCFH-DA. The cells were incubated with 10 *μ*mol/L DCFH-DA at 37°C during the last 20 min of Con, Bup, GB, or GB + Bup treatment. Treated and DCFH-DA-stained cells were washed 3 times in PBS, harvested, and resuspended in PBS. Fluorescent signal intensity was determined by flow cytometry to estimate relative ROS accumulation.

### 2.7. Mitochondrial Membrane Potentials Assay

Mitochondrial membrane potential (*ψ*m) depolarization, an early event in the mitochondrial apoptosis cascade, was measured fluorometrically using JC-1. Briefly, cells cultured in 6-well plates and treated as described for the ROS measurement were incubated with JC-1 staining solution (5 *μ*g/mL) at 37°C for 20 min and rinsed twice with PBS. Mitochondrial membrane potential was estimated by measuring the fluorescence ratio of free JC-1 monomers (green) to JC-1 aggregates in mitochondria (red) by dual emission fluorescence microscopy (Nikon ECLIPSE TE2000-u, Japan) and flow cytometry. Mitochondrial depolarization is indicated by an increase in the proportion of cells emitting green fluorescence.

### 2.8. Isolation of Mitochondrial

Mitochondria were isolated from SH-SY5Y cells cultured in 6-well plates and treated as described for ROS measurements. After rinsing twice in PBS, cells were harvested, centrifuged at 600 ×g for 5 min at 4°C, and then homogenized in 1 mL mitochondria isolation reagent until 50% of the cells were lysed. Homogenates were centrifuged at 600 ×g for 5 min at 4°C to remove large debris and unlysed cells. The supernatant containing mitochondria then transferred to another centrifuge tube, and mitochondria precipitated by centrifugation at 11,000 ×g for 10 min at 4°C. After centrifugation, the pellet was resuspended in mitochondrial storage solution.

### 2.9. Measurement of Respiratory Complex I and Complex III Activities

The activities of respiratory chain complexes I and III were determined according to the methods as described by Zhang et al. [24]. All assays were performed at 25°C in a final volume of 1 mL using a spectrophotometer. To release complexes from the mitochondrial membrane, isolated mitochondria were subjected to three freeze-thaw cycles (25°C to −25°C) in hypotonic media (25 mmol/L potassium phosphate, 5 mmol/L MgCl_2_, pH 7.2) before activity measurements. The enzyme activity was expressed in nanomolars per minute per milligram protein.

### 2.10. Western Blotting

Cells were incubated as described for the ROS measurements, harvested, and lysed in lysis buffer. After centrifugation, the soluble protein concentration in the supernatant was determined by a BCA Protein Assay Kit (Beyotime, China). Protein samples were separated by sodium dodecyl sulfate-polyacrylamide gel electrophoresis (20 *μ*g/lane), electrotransferred to polyvinylidene difluoride (PVDF) membranes. Membranes were blocked with 5% nonfat dry milk in Tris-buffered saline and then immunoblotted with anti-Grp78 (1 : 500), anti-caspase-12 (1 : 500), anti-cleaved caspase-3 (1 : 500), anti-HtrA2 (1 : 500), or anti-GAPDH antibody (1 : 1000, as the gel loading control) overnight at 4°C. All antibodies were diluted in Tris-HCl-buffered saline containing 5% nonfat dry milk and 0.1% Tween-20. After rinsing, immunolabeled membranes were incubated with horseradish peroxidase (HRP) conjugated anti-rabbit immunoglobulin (1 : 1000) for 1 h. Specific proteins were detected by enhanced chemiluminescence and exposure to X-ray film. Bands were quantified by scanning the films. The expression levels of Grp78, caspase-12, cleaved caspase-3, and HtrA2 protein were normalized to GAPDH.

### 2.11. Quantitative Real Time PCR (qRT-PCR)

To investigate the effect of bupivacaine on ER stress, we examined Grp78 and caspase-12 mRNA expression levels by qRT-PCR. Total RNA was isolated using an RNA Isolation Kit (Qiagen, USA) according to the manufacturer's instructions. DNase I (TAKARA, Japan) was used to remove DNA from total RNA. cDNA was synthesized using a cDNA Synthesis Kit (Promega, USA), and the Maxima SYBR Green qPCR Master Mix (2X) (Fermentas, USA) was used to quantify gene expression. Conditions for amplification and quantification included initial denaturing (50°C for 2 minutes and 95°C for 10 minutes) followed by 40 cycles of 2 amplification stages (95°C for 15 seconds and 60°C for 1 minute) for primer annealing and elongation. A dissociation stage (95°C for 15 seconds, 60°C for 1 minute, and 95°C for 15 seconds) was added at the end of amplification stage to ensure that a single amplicon was produced and to validate the primer pairs. Reactions were performed in triplicate. Relative expression levels of caspase-12 and Grp78 mRNA were quantified using the 2-ΔΔCT method [25, 26] and 18 srRNA as the normalizing gene. The primers used are listed in Table 1.

### 2.12. Transmission Electron Microscopy (TEM)

Neuroblastoma cells treated as described for the ROS measurements were harvested, washed once with PBS, fixed in 2.5% glutaraldehyde at 4°C for 1 h, postfixed in 1% osmic acid for 30 min, and stained with lead uranium. Cell ultrastructure was observed under a transmission electron microscope (Hitachi-600, Japan).

### 2.13. Statistical Analysis

All values are expressed as means ± SD. Multiple comparisons between groups were analyzed by one-way ANOVA. LSD was performed as post hoc analysis for multiple comparisons between groups. A *P* < 0.05 was considered significant.

## 3. Results 

### 3.1. Bupivacaine Reduced Cell Viability

**Figure 1:**
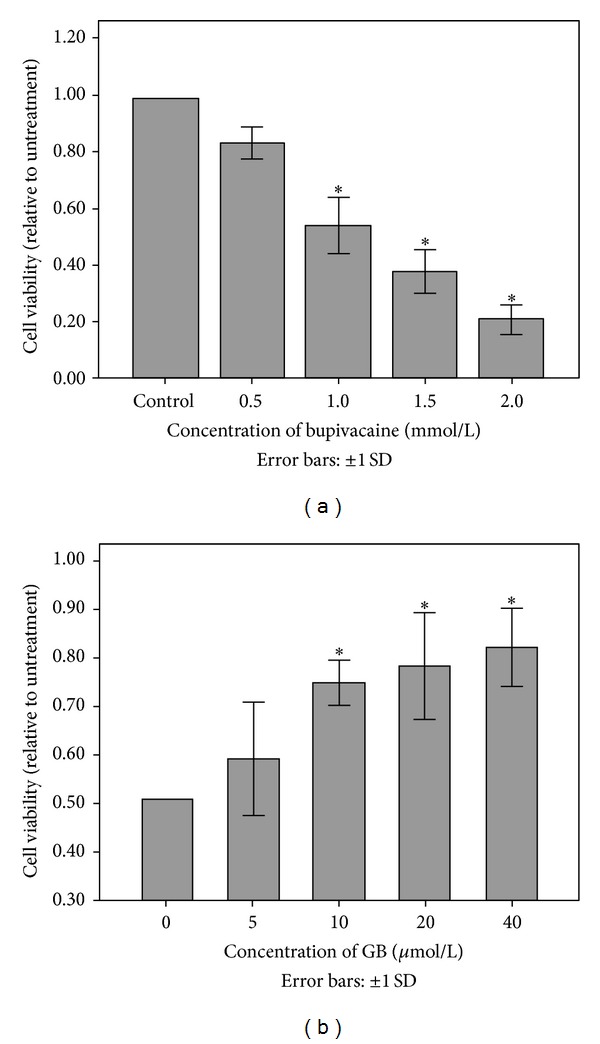
Proliferation effects of bupivacaine on SH-SY5Y cells and GB weakened bupivacaine-induced cell injury in SH-SY5Y cells. (a) Cells were incubated in the presence or absence of various concentrations of bupivacaine for 24 h (*P < 0.05 versus control group). Cell growth was determined by the MTT assay. (b) Cell viability was decreased by treatment with 1 mmol/L bupivacaine for 24 hours. Decreased viability was inhibited by GB pretreatment for 6 hours, except for cells treated with 5 *μ*mol/L GB (*P < 0.05 versus nonpretreated group). Each data point represents the mean ± SD of 6 separate experiments.

The effect of bupivacaine on the viability of SH-SY5Y neuroblastoma cells was first examined using the by MTT assay. Bupivacaine (1, 1.5, 2 mmol/L) significantly reduced viable cell number compared to controls (Figure 1(a)). We then estimated viable cell number at multiple time points during treatment with 1 mmol/L bupivacaine, the LD50 measured in a previous study [23]. Compared to controls, bupivacaine reduced viable cell number at all time points between 24 and 48 h. Thus, bupivacaine reduced SHSY5Y cell proliferation, induced cell death, or both.

### 3.2. GB Attenuated Cell Toxicity Induced by Bupivacaine

In our pilot experiment, treatment with 5–40 *μ*mol/L GB for 6 h did not affect cell proliferation. The antiproliferative or cytotoxic effect of 1 mmol/L bupivacaine was then compared between GB-pretreated (5–40 *μ*mol/L) and GB-naïve cultures using the CCK-8 assay. Cell counts were higher at all GB doses except at 5 *μ*mol/L, the lowest dose tested (Figure 1(b)). To determine if GB actually protected SHSY5Y cells against bupivacaine-mediated cytotoxicity, apoptosis was examined by flow cytometry.

### 3.3. GB Attenuated Bupivacaine-Induced SH-SY5Y Cell Apoptosis

**Figure 2:**
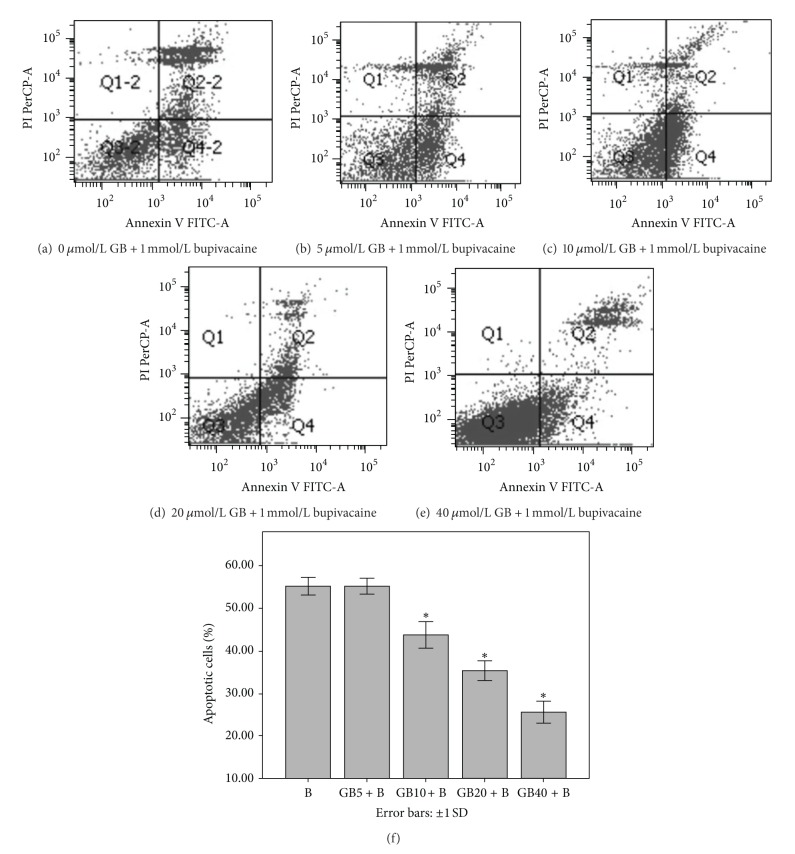
GB pretreatment decreased the number of apoptotic cells induced by bupivacaine. ((a)–(e)) Cells were treated with 0, 5, 10, 20, and 40 *μ*mol/L GB for 6 hours, respectively, prior to treatment with 1 mmol/L bupivacaine for 24 h. (f) Summarized data show apoptotic rate as detected by flow cytometry. Data represented are the mean ± SD of 6 separate experiments (*P < 0.05 versus 0 *μ*mol/L GB).

Ginkgolide B pretreatment decreased bupivacaine-induced apoptosis as evidenced by reduced Annexin V+/PI− and Annexin V+/PI+ cell numbers (representing early or late apoptosis, resp.) at 40 *μ*mol/L GB (Figure 2). The reduction was similar for both Annexin V+/PI− and Annexin V+/PI+ cell populations, indicating that GB blocked the initiation of apoptosis.

### 3.4. GB Attenuated ROS Production Induced by Bupivacaine

**Figure 3:**
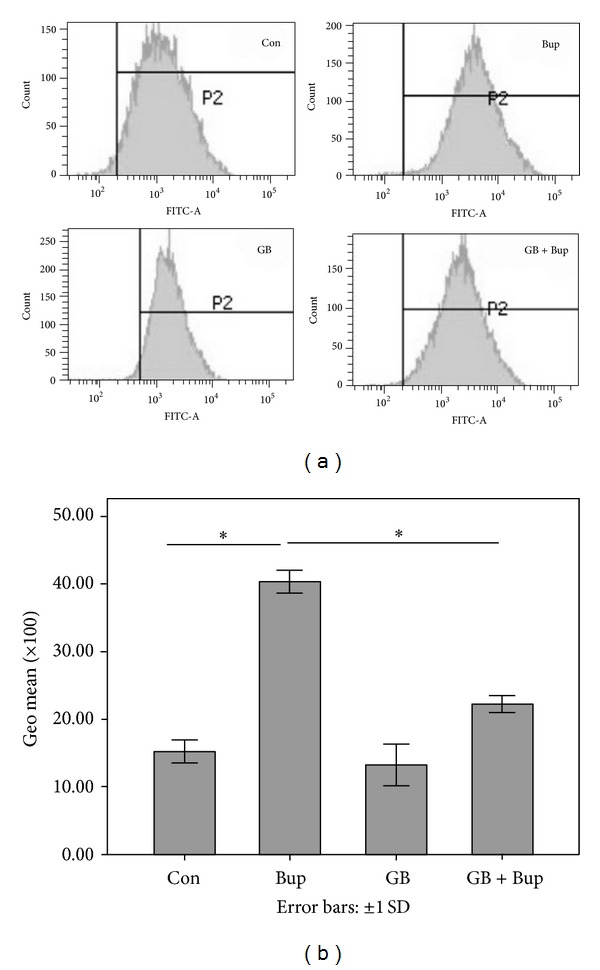
The levels of reactive oxygen species were measured by flow cytometry. GB pretreatment decreased ROS overproduction induced by bupivacaine. Summarized data shows the Geo mean of ROS as detected by flow cytometry. Data represented are mean ± SD of 6 separate experiments (*P < 0.01).

Treatment with 1 mmol/L bupivacaine increased the intracellular ROS accumulation, indicated by DCFH-DA fluorescence, while GB pretreatment significantly reduced the ROS-dependent fluorescent signal (Figure 3). These results suggest that GB acts to preserve mitochondrial function, elevates endogenous antioxidant capacity, and (or) possesses inherent antioxidant activity.

### 3.5. GB Inhibited Mitochondrial Depolarization Induced by Bupivacaine

**Figure 4:**
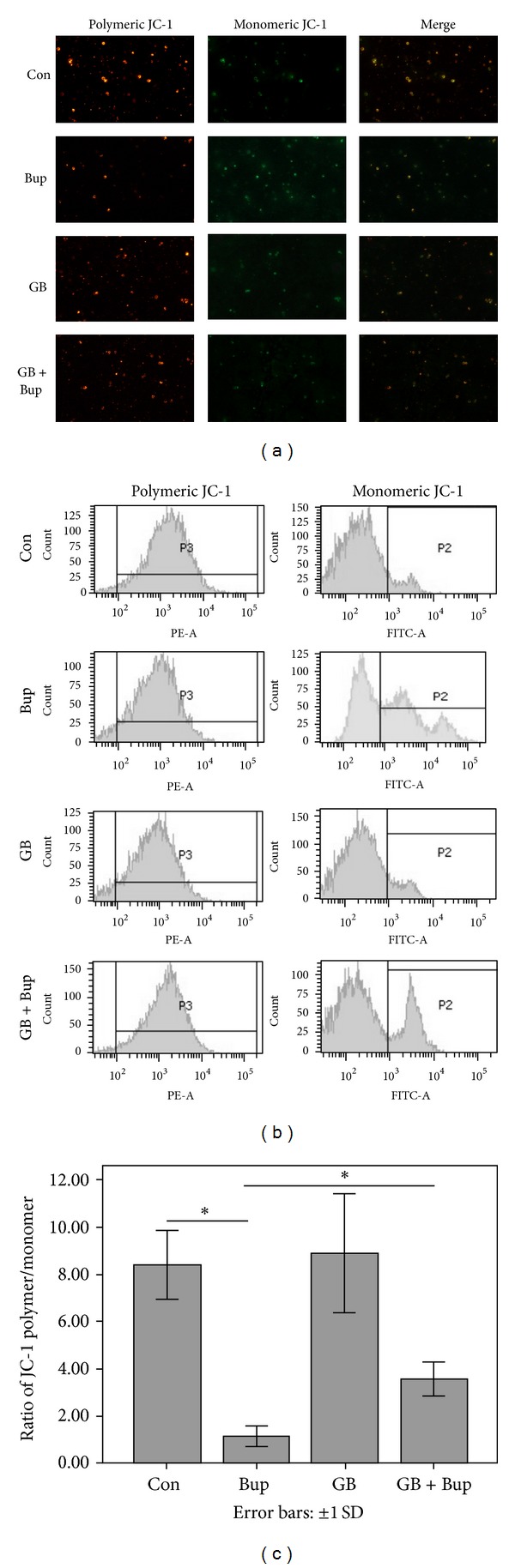
GB attenuated the bupivacaine-induced decline of mitochondrial membrane potential (Δ*ψ*m). SH-SY5Y cells were treated with bupivacaine for 24 hours in the presence or absence of GB. (a) SH-SY5Y cells were observed using fluorescent microscopy. (b) Δ*ψ*m were detected by flow cytometry (c) Δ*ψ*m expressed as the ratio of red fluorescence over green fluorescence. Data represented are the mean ± SD of 6 separate experiments (*P < 0.01).

The mitochondrial membrane potential (*ψ*m) is correlated with functional activity, while loss of *ψ*m (depolarization) is indicative of mitochondrial uncoupling and is an early sign of apoptosis. We estimated *ψ*m in SH-SY5Y cells by the shift in JC-1 fluorescence (from red to green). Exposure to 1 mmol/L bupivacaine resulted in *ψ*m dissipation, while GB pretreatment preserved *ψ*m during bupivacaine exposure (Figure 4). These results suggest that GB may prevent apoptosis by preserving mitochondrial function and by preventing activation of mitochondrial-dependent apoptosis.

### 3.6. Preservation of Mitochondrial Respiratory Complex I and III Activity by GB

**Figure 5:**
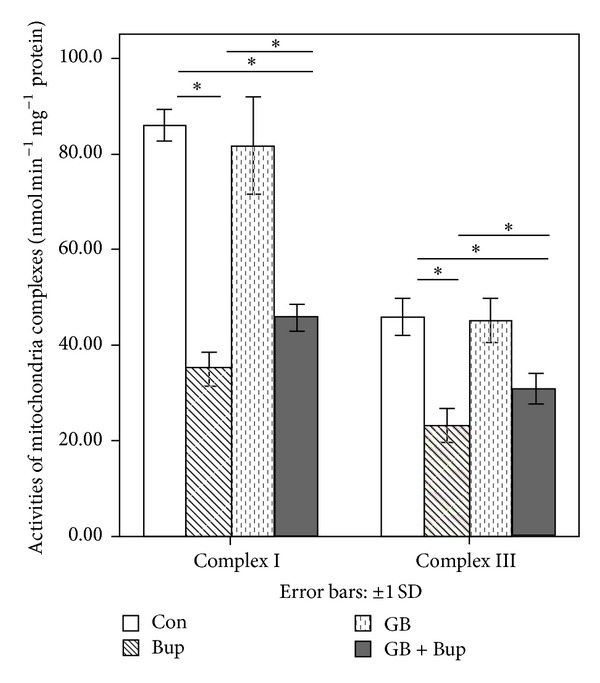
GB attenuated bupivacaine-induced decrease in the activity of mitochondrial complexes I and III. Experiments were repeated 6 times, and the data were presented as the mean ± SD (*P < 0.01).

The activities of mitochondrial complexes I and III were significantly reduced by bupivacaine, suggesting potential loss of oxidative phosphorylation. Again, disruption of mitochondrial function was reversed by GB pretreatment (Figure 5).

### 3.7. The Level of Cleaved Caspase-3 and HtrA2

**Figure 6:**
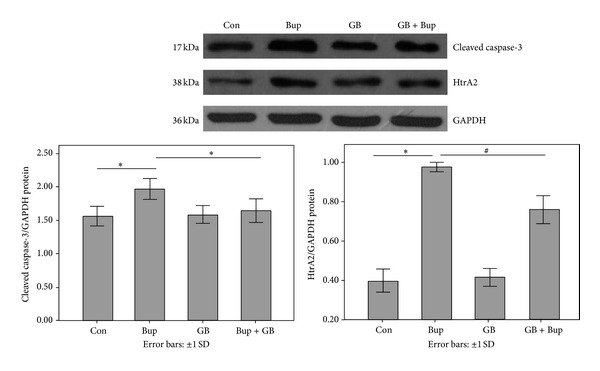
Htra2 induction and cleaved caspase-3 activation were detected by Western blot. Experiments were repeated three times, and the data were presented as mean ± SD (*P < 0.01; ^#^ *P* < 0.05).

Mitochondrial uncoupling and *ψ*m depolarization are associated with a dramatic increase in mitochondrial inner membrane permeability and the release of factors (e.g., cytochrome c and HtrA2) that initiate or regulate apoptotic pathways. Western blots (Figure 6) revealed significantly elevated cleaved (activated) caspase-3 and HtrA2 expression in bupivacaine-treated cultures compared to controls, an effect that was suppressed by GB pretreatment.

### 3.8. GB Reduced ER Stress Induced by Bupivacaine

**Figure 7:**
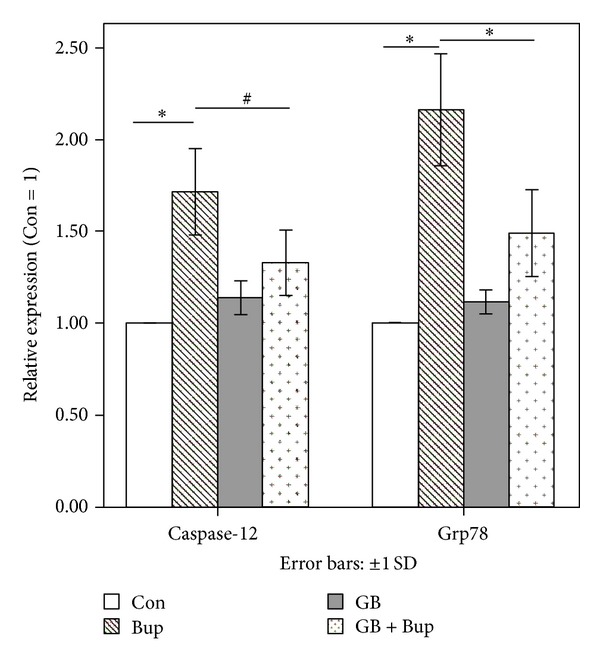
Grp78 and caspase-12 mRNA expression as detected by qRT-PCR. Experiments were repeated three times, and the data were presented as the mean ± SD (*P < 0.01, ^#^ *P* < 0.05).

**Figure 8:**
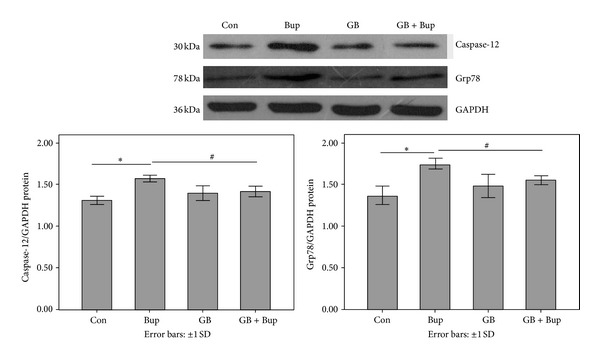
Grp78 and caspase-12 expression representing ER stress as detected by Western blot. Experiments were repeated three times, and the data were presented as the mean ± SD (*P < 0.01, ^#^ *P* < 0.05).

Expression of Grp78 and caspase-12 is indicative of ER stress. Quantitative RT-PCR and Western blots revealed enhanced Grp78 and caspase-12 mRNA (Figure 7) and protein expression levels (Figure 8) in BG-naïve bupivacaine-treated cells compared to controls, responses that were reversed by GB pretreatment.

### 3.9. Morphological Changes of Cells

**Figure 9:**
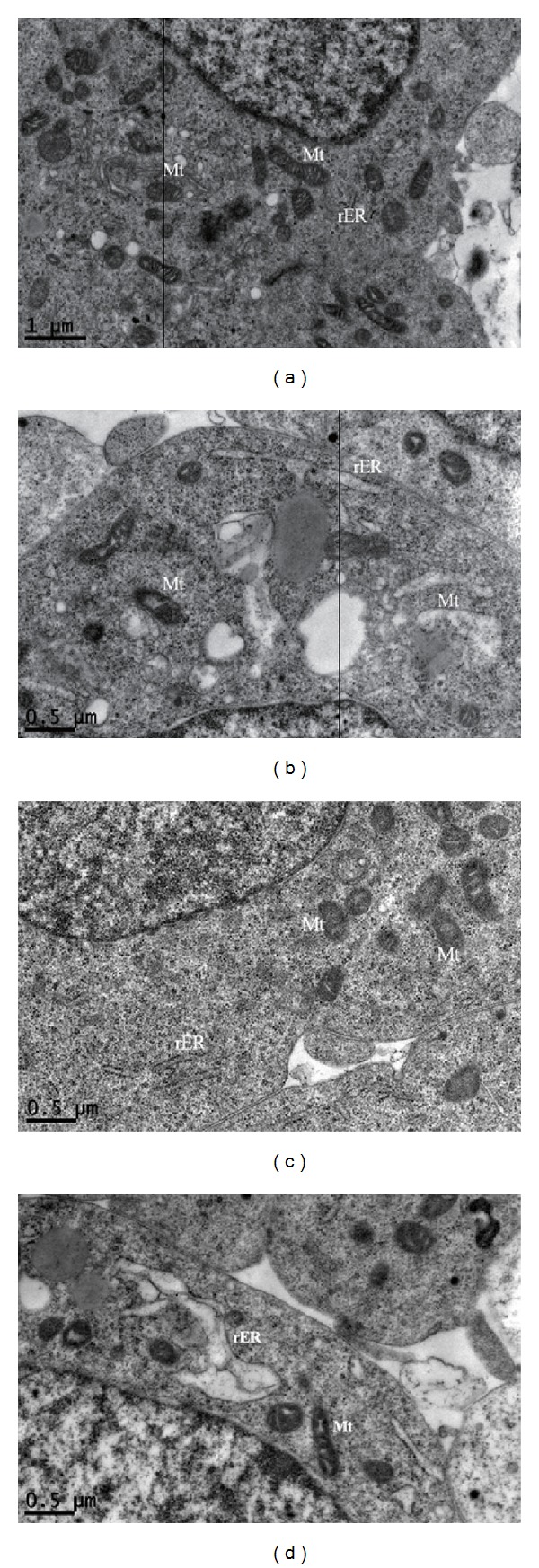
Morphologic changes of SH-SY5Y cells. (a) Cells in the control group retained a normal ultrastructure; (b) cells in the Bup group contained degranulated rER, swollen Mt, and hazy mitochondrial structures; (c) treatment with GB resulted in and showed a nearly normal structure; (d) pretreatment with GB represented slight expansion of ER.

Normal healthy SH-SY5Y cells were round and regular, with typically shaped ER and mitochondrial membranes in TEM images (Figure 9(a)). After exposure to bupivacaine for 24 h, the ER appeared swollen and degranulated, while mitochondria were swollen with loss of internal membrane structure (Figure 9(b)). Cells treated with 40 *μ*mol/L GB showed a nearly normal ultrastructure (Figure 9(c)), indicating that GB had little endogenous toxicity or physiological effects on ER or mitochondrial function. Cells pretreated with GB prior to bupivacaine exposure resembled controls at the ultrastructural level, with only slight expansion of the ER (Figure 9(d)). This preservation of ER and mitochondrial structure strongly suggests that GB protected SH-SY5Y cells against bupivacaine-induced mitochondrial and ER damage.

## 4. Discussion

Ginkgolide B (GB), an active component of the traditional medicinal herb Ginkgo biloba, protected SH-SY5Y cells from bupivacaine-induced injury. Pretreatment with 40 *μ*mol/L GB suppressed bupivacaine-induced mitochondrial depolarization, mitochondria complex I and III inhibition, ROS accumulation, ER stress, and apoptosis. These results implicate mitochondrial dysfunction and ER stress in bupivacaine-induced apoptosis and highlight GB as a potential neuroprotectant against bupivacaine toxicity through its antioxidant property.

The therapeutic time window is critical in defining the potential clinical utility of any neuroprotective agent. Ginkgolide B has been shown to exert significant protective effect in cerebral ischemia injury up to 2 h following intravenous administration after reperfusion in rat [27]. In our pilot experiment, pretreatment with 5–40 *μ*mmol/L GB for 2 h, 4 h could not protect SH-SY5Y cells from bupivacaine neurotoxicity, which is different from other people's previous research. Only 6 h duration of pretreatment with GB conferred protective effect; therefore, 6 h treatment protocol was used in our study.

Oxidoredox homeostasis is essential for cellular survival. Overproduction of ROS leads to oxidative stress and plays an important role in the process of apoptosis in many cell types [28], which can be ameliorated by endogenous and exogenous antioxidants. Bupivacaine was shown to induce ROS generation in SH-SY5Y cells [6], while GB reduced ROS levels *in vivo *[22], suggesting that GB may protect against bupivacaine toxicity by suppressing ROS accumulation. Bupivacaine did substantially increase ROS, a major initiator of apoptosis [29], while preincubation with GB suppressed ROS accumulation and many of the biochemical and morphological signs of oxidative stress. To investigate the potential reasons for increased ROS production, we measured the activities of mitochondrial complexes I and III, the main generators of ROS [30]. The activity of both complexes decreased after bupivacaine treatment, while GB pretreatment partially reversed this effect. By preserving oxidative phosphorylation, GB maintained *ψ*m and decreased ROS production associated with mitochondrial uncoupling. Aside from mitochondrial dysfunction, however, ROS may also be generated by calcium-dependent protease activity, nNOS, and acidosis, the contributions of which were not examined and warrant further study as possible mechanisms of bupivacaine toxicity.

Besides energy production via the electron transport chain, mitochondria are responsible for several other important cellular functions, including the initiation and regulation of apoptosis [31]. Local anesthetics may dissipate *ψ*m and activate caspases, leading to apoptotic cell death [6, 32]. In our study, apoptotic cell death induced by bupivacaine was associated with *ψ*m depolarization, and both bupivacaine-induced apoptosis and *ψ*m dissipation were attenuated by GB. The HtrA2 protein is a serine protease that acts as a proapoptotic factor following release from the mitochondrial matrix through large nonselective pores (permeability transition pores, mPTPs) that can be opened by overproduction of ROS [33, 34]. Release of mitochondrial HtrA2 into the cytoplasm was inhibited by the caspase inhibitor z-VAD-fmk [34, 35], suggesting that caspase activation may precede and possibly induce HtrA2 release. Increased cleaved caspase-3 is associated with mitochondria-dependent apoptosis following sustained loss of *ψ*m [36]. Thus, bupivacaine likely induced apoptosis by reducing mitochondria complex activity, leading to overproduction of ROS, collapse of the mitochondrial membrane potential, release of proapoptotic factors from the mitochondrial matrix, and subsequent caspase-3 activation.

ES stress may activate alternate apoptotic pathways or exacerbate mitochondria-dependent apoptosis. The ER is critical for protein synthesis and folding, lipid and sterol synthesis, and calcium homeostasis. Stressors such as hypoxia, glucose deprivation, and calcium depletion from the ER lumen lead to ER dysfunction [37], resulting in cellular calcium dysregulation, protein misfolding and aggregation, and activation of proapoptotic effectors such as caspase-12. Bupivacaine caused ER stress as evidenced by elevated caspase-12 and Grp78 expression, and this stress may have resulted from ROS accumulation as reported by Takahashi et al. [17].

Grp78 is a well-characterized indicator of UPR activation (the unfolded protein response) and a critical protectant against ER stress by preventing protein aggregation [38]. However, when the ER stress is severe or prolonged, the increase in Grp78 is no longer sufficient to prevent apoptosis, and the UPR switches from a cytoprotective to a proapoptotic response involving activation of specific effector proteins such as caspase-12, which is activated only by ER stress-initiated apoptotic pathways [37]. In accordance with previous studies [39, 40], Grp78 expression was upregulated in parallel with caspase-12, indicative of ER stress and ER stress-specific apoptosis. Ginkgolide B inhibited the overexpression of Grp78 and caspase-12, suggesting that suppression of cell death resulted from disruption of both mitochondrial and ER-dependent apoptotic pathways. This conclusion was further corroborated by TEM images showing reduced organelle swelling and maintenance of ER and mitochondrial membrane integrity in cells pretreated with GB prior to bupivacaine.

Some limitations of this study should be noted. First, we examined doses of bupivacaine (1 mmol/L or 0.03%) that are not clinically relevant, as local injections often use 0.25% or 0.5%, although the duration of exposure was greatly prolonged in this study. Second, these *in vitro* results from transformed neuroblastoma cells may not be applicable to neurons *in vivo*. Nonetheless, previous results have demonstrated morphological signs of oxidative stress and apoptosis following local anesthetic administration *in vivo*, and exogenous antioxidants (like GB) have well-established neuroprotective efficacy.

In summary, the current study suggests that bupivacaine elicits ROS production, which in turn triggers mitochondrial depolarization, mitochondria-dependent apoptosis, and ER stress. These pathological responses were reduced or ameliorated by pretreatment with ginkgolide B. These results provide novel insights into the molecular mechanisms underlying the neurotoxicity of bupivacaine and highlight GB as a prototype treatment for the neurotoxicity elicited by this class of local anesthetics.

## Figures and Tables

**Table 1:** qRT-PCR primers.

Gene	Primers
18srRNA	Forward: 5′-CCT GGA TAC CGC AGC TAG GA-3′
	Reverse: 5′-GCG GCG CAA TAC GAA TGC CCC-3′

GRP78	Forward: 5′-TGC AGC AGG ACA TCA AGT TC-3′
	Reverse: 5′-CGC TGG TCA AAG TCT TCT CC-3′

Caspase-12	Forward: 5′-GGA GAA AGA GAG GCG AAC AT-3′
	Reverse: 5′-CCT GGA TAC CGC AGC TAG GA-3′
